# The Psychological and Quality of Life Impacts on Women in Hong Kong during the COVID-19 Pandemic

**DOI:** 10.3390/ijerph18136734

**Published:** 2021-06-23

**Authors:** Maria Shuk Yu Hung, Stanley Kam Ki Lam, Liliane Chui King Chan, Sisi Pui Shan Liu, Meyrick Chum Ming Chow

**Affiliations:** 1 School of Nursing, Tung Wah College, Hong Kong, China; meyrickchow@twc.edu.hk; 2 LKS Faculty of Medicine, School of Nursing, The University of Hong Kong, Hong Kong, China; stanley.lam@hku.hk; 3 Hong Kong Federation of Women’s Centres, Hong Kong, China; liliane.cck@gmail.com (L.C.K.C.); sisi.liu@womencentre.org.hk (S.P.S.L.)

**Keywords:** COVID-19 pandemic, psychological impacts, quality of life, Hong Kong, women, depression, anxiety, stress, self-efficacy

## Abstract

The coronavirus (COVID-19) pandemic has caused a global health crisis. The adverse impacts on Asian women, including those in Hong Kong, are substantial. This cross-sectional online study examined the impacts of COVID-19 on Hong Kong women, including psychological effects, self-belief in coping, and quality of life, and was conducted over 4 weeks from July to August 2020. Females aged over 18, living in Hong Kong, and that could read Chinese, were included. Among 417 participants, 50.8% were aged below 50, 66.7% were married, 57.1% were caregivers, 61.4% had a family income of <USD 2600, and 70.3% attained higher secondary education or above. The results show that 32.2%, 42.4%, and 44.9% of participants had negative emotions of stress, anxiety, and depression. There are significant negative correlations between emotional state and different aspects of quality of life, but positive correlations between general self-efficacy and different aspects of quality of life. COVID-19 induced significant psychological and quality of life impacts on females in Hong Kong. The policymakers, healthcare professionals, and social support organizations should establish appropriate strategies and policies to support women during the COVID-19 pandemic or similar future crises.

## 1. Introduction

The coronavirus (COVID-19) pandemic has caused a global health crisis and has had catastrophic impacts on humans since late 2019. Up to the end of May 2021, there have been about 170 million confirmed cases of COVID-19 and more than 3.5 million deaths globally [1]. In addition to the pandemic’s tremendous physical negative health impacts on humans, studies on the mental health crisis reported that emotional distress is ubiquitous worldwide [2,3,4,5,6]. These studies stated that COVID-19 induced negative psychological responses among general populations. A systematic review summarized the common adverse symptoms, such as depression, anxiety, and psychological distress/stress symptoms [7]. In general, the associated risk factors or predictive factors include a younger age group (≤40 years), poor self-rated health, the presence of chronic illness, lower education levels, lower household income, quarantine status, female gender, etc. [7]. Although the literature suggests that men have a higher proportion of poor clinical outcomes and mortality than women due to COVID-19 [8,9], women experienced more negative emotions and psychological impacts than men [5,7,10]. For instance, women had a higher risk of anxiety and depression than men [10,11,12,13]. In a study conducted in China, women were found to be more vulnerable and had three times the level of anxiety than men due to women’s social, cultural and family roles of caregiving [5]. Another review stated the intensified risk of specific adverse health outcomes that affect or worsen women’s health during COVID-19, particularly among healthcare workforce, gender-based violence, and mental health perspectives [14].

In consideration of traditional Hong Kong’s specific social and cultural context, Chinese women still have the primary responsibilities of housework and childrearing, limiting their personal and professional growth options [15]. Women in Hong Kong are more educated nowadays and they join the labor market in a similar way to men. However, women’s pressure in society is immense due to personal, familial, and societal responsibilities [15]. This can be unfavorable to their mental health and induce undesirable health consequences for women in Hong Kong during the COVID-19 pandemic. Concerning the literature and evidence that public health emergencies affect women severely, this study aimed to assess the impacts of COVID-19 on Hong Kong women, including psychological effects, self-belief in coping, and quality of life. This study is the first study to examine the impacts on women in Hong Kong during the pandemic. The results could have implications for healthcare professionals, social support organizations, and policymakers in creating appropriate strategies, interventions, and policies in supporting women during the pandemic and similar public health emergencies in the near future.

## 2. Materials and Methods

### 2.1. Design

The study was a quantitative study using a cross-sectional survey to assess the impacts of COVID-19 on Hong Kong women.

### 2.2. Instrument and Outcome Measures

A self-reported online questionnaire comprising 5 parts was used for data collection. The first part included personal demographics, such as age, marital status, education attainment, employment, and caregiving roles. In the second part, since no validated assessment tools were available in the early stage of the COVID-19 pandemic, the researchers developed a simple 10-point rating scale (ranged from 1–10) to produce initial estimates of how the respondents rate their (1) fear of, (2) worries about, and (3) perceived influences of COVID-19, of which higher scores indicate increasing severity. The other parts were three well-validated scales adopted to examine women’s psychological impacts, self-efficacy, and quality of life during the COVID-19 pandemic in Hong Kong.

The Chinese Depression Anxiety Stress Scales (DASS 21) measure emotional states, comprising three constructs: depression, anxiety, and stress [16,17]. It has 21 items with a 4-point Likert-type scale ranging from 0 (did not apply to me at all) to 3 (applied to me very much, or most of the time) [16]. Higher scores indicate more negative emotional states. Typical items include ‘I found it difficult to relax’, ‘I experienced breathing difficulty’, ‘I felt life was meaningless’, etc., to assess different constructs [16]. The General Self-Efficacy (GSE) scale (Chinese version) was selected to determine the women’s self-belief in coping with COVID-19 [18]. The GSE scale was commonly used to evaluate the optimistic self-beliefs in dealing with various difficult life situations [18,19]. The scale includes ten items with a 4-point Likert-type scale ranging from 1 (Not at all true) to 4 (Exactly true). Higher scores indicate higher self-efficacy. Example items include, ‘I can always manage to solve difficult problems if I try hard enough’, and ‘I can remind calm when facing difficulties because I can rely on my coping abilities’. The World Health Organization Quality of Life Scale Abbreviated Version (WHOQOL-BREF) was developed to measure the quality of life [20]. In this study, it was used to examine the participants’ overall quality of life and general health and the four broad domains, including physical health, psychological health, social relationships, and the environment. The scale is composed of 26 questions with a 5-point Likert-type scale, and the higher total scores indicate a better quality of life [20]. All three of these adopted Chinese scales have been well-validated in China [17,21,22]. Before study implementation, the authors obtained approval in using these scales as required.

### 2.3. Ethical Considerations

The study was conducted according to the guidelines of the Declaration of Helsinki and approved by the Research Ethics Sub-Committee of the principal investigator’s institution. The study’s purpose and detailed information about it were provided to the potential participants for consideration. They had the right to refuse participation and could refuse to respond to any items in the questionnaire. Electronic consent was obtained from each subject before data collection. All the data were kept confidential. The anonymity of the participants was guaranteed.

### 2.4. Subjects and Recruitment

The potential subjects were invited through the Hong Kong Federation of Women’s Centres. This is a non-government organization established for 4 decades with five centers that are located in different regions, including Hong Kong Island, Kowloon and New Territories, to provide services for the whole of Hong Kong. The inclusion criteria were those Chinese females aged over 18, living in Hong Kong, who could read and understand Chinese. The exclusion criteria were all males, females aged below 18, and those who could not read Chinese. Purposive and snowball sampling was employed through the five centers in different locations to invite their members to join the study. Then, the members were encouraged to forward the link to their female friends. To determine the sample size for this study, power analysis for multiple linear regression was conducted with the following input parameters: a power of 0.90, an effect size of 0.15, 5 predictors and covariates at a confidence level of 95%. Using G*Power 3.1.9.6, the required sample size is 73 [23]. With 417 observations in our study, the sample size was adequate for this regression. It also satisfied the rule of thumb of 20 observations per co-variate of interest.

### 2.5. Data Collection and Analysis

Data were collected online for four weeks from July to August 2020 during the ‘third wave’ of COVID-19 in Hong Kong. After receiving the returned questionnaires, the uncompleted questionnaires were excluded. The IBM SPSS-Statistics 26 package (Armonk, NY, USA) was used for the data analysis. Cronbach’s alpha suggests that these scales have high internal consistency (DASS 21 = 0.95; WHOQOL-BREF = 0.95; General self-efficacy scale = 0.95). Descriptive statistics were used to present the participants’ demographics. Inferential statistics such as Pearson correlations were used to study the relationship between emotional states, general self-efficacy and four domains of quality of life. The associations between participants’ characteristics and emotional states, general self-efficacy and four domains of quality of life were examined using bivariate and multivariable regression analyses.

## 3. Results

A total of 417 out of 469 women answered the online questionnaire completely. The snowball sampling and online questionnaire methodology used in this study did not allow to calculate the attrition rate. Among these 417 participants, half were aged <50 (50.8%, *n* = 212), and about seventy percent of participants (70.3%, *n* = 293) attained a senior secondary education or above (high education). Two thirds of participants (66.7%, *n* = 278) were married, and around three fifths (61.4%, *n* = 256) had a family monthly income of <USD 2600. More than half of them (57.1%, *n* = 238) had caregiving responsibilities.

### 3.1. The Scores of Fear, DASS21, GSE, and WHOQOL-BREF during COVID-19

Using the simple 10-point rating scale to assess how the participants rate their fear, worries, and perceived influences of COVID-19, the mean scores were 7.59 (SD = 2.10), 7.79 (SD = 2.01), and 8.10 (SD = 1.84), respectively, which shows that the pandemic induced severe psychological impacts on our participants.

Using the DASS21, the participants’ mean scores of stress, anxiety, and depression were 12.35 (SD = 8.82), 7.71 (SD = 7.76), and 9.35 (SD = 8.40), respectively. Based on the normative data and recommended cut-off scores of DASS by Lovibond and colleagues [16], 11.7%, 12%, and 8.5% of participants were classified as having mild, moderate, and severe/extremely severe stress levels, respectively. For anxiety levels, 6.2%, 21.1%, and 15.1% of participants had mild, moderate, and severe/extremely severe anxiety, respectively. In addition, 17.3%, 18.2%, and 9.4% of participants had mild, moderate, and severe/extremely severe depression, respectively. In sum, 32.2%, 42.4%, and 44.9% of the study participants had negative emotions of stress, anxiety, and depression, respectively, during the ‘third wave’ of the COVID-19 pandemic in Hong Kong.

For the participants’ self-belief in coping with various challenging life situations, their GSE mean score is 22.95 (SD = 6.04). Regarding their mean scores for the four domains of WHOQOL BREF, physical health is 14.07 (SD = 2.63), psychological health is 12.72 (SD = 2.87), social relationships is 12.90 (SD = 2.54), and the environment is 12.24 (SD = 2.81). Table 1 presents the participants’ mean scores and standard deviations for the scales of DASS21, GSE, and WHOQOL-BREF. Additionally, Table 2 shows the descriptive statistics of demographic variables, emotional states, general self-efficacy, and four domains of the quality of life.

### 3.2. The Correlations between Emotional States, General Self-Efficacy, and the Four Domains of Quality of Life

There are significant negative correlations between emotional states and the quality of life of our study participants. For example, anxiety is negatively correlated with physical health (r = −0.62, *p* < 0.01), psychological health (r = −0.61, *p* < 0.01), social relationships (r = −0.48, *p* < 0.01) and environment (r = −0.50, *p* < 0.01); depression is negatively correlated with physical health (r = −0.60, *p* < 0.01), psychological health (r = −0.68, *p* < 0.01), social relationships (r = −0.46, *p* < 0.01) and environment (r = −0.47, *p* < 0.01). In contrast, general self-efficacy is positively correlated with physical health (r = 0.58, *p* < 0.01), psychological health (r = 0.69, *p* < 0.01), social relationships (r = 0.60, *p* < 0.01) and environment (r = 0.57, *p* < 0.01). Table 3 presents the correlations between emotional states, self-efficacy and quality of life.

### 3.3. The Associations between Demographic Variables and Emotional States, General Self-Efficacy, and the Four Domains of Quality of Life

Bivariate regression models were used to examine the associations between participants’ characteristics and the dependent variables of emotional states, general self-efficacy and the four domains of quality of life. The results are presented in Table 4. Age, education level, being a caregiver or not, and monthly family income were significantly associated with most of the dependent variables, whereas marital status was not associated with the dependent variables.

Multivariable regression analyses, after controlling for participants’ age, education level and marital status, found that being a caregiver was associated with a significantly higher level of stress, lower general self-efficacy, physical health, psychological health, and being less satisfied with one’s social relationship and living environment. Those with a monthly family income less than USD 2600 reported significantly higher stress, anxiety, and depression, lower general self-efficacy and physical health, and being less satisfied with their living environment. Table 5 shows the results of multivariable regression analyses.

## 4. Discussion

This study demonstrated the significant psychological and quality of life impacts on women during the ‘third wave’ of the COVID-19 pandemic in Hong Kong from July to August, which was most severe among the several waves of the pandemic in Hong Kong. Additionally, all these psychological and quality of life impacts are overarching and interrelated.

### 4.1. Fear, Worries, and Risk Perception of COVID-19

Previous research suggested a positive correlation between fear of COVID-19 and change in public health behaviors, enhancing the citizens’ compliance with public health behaviors, such as maintaining social distancing and hand hygiene [13,24]. A survey of 1715 citizens was conducted right after the first confirmed case of COVID-19 in late January 2020 in Hong Kong [25]. The results show that the respondents had mild anxiety, high risk perception, were alert to COVID-19, implemented self-protective measures and that the female respondents were more adherent to the social distancing policy. In addition, the sudden increase in demand for face masks and personal protective items and inadequate supply or shortage of these items globally caused extensive fears, with panic over masks and food hoarding in Hong Kong [2,26]. However, a sudden rebound of the ‘third wave’ in mid-July 2020 caused an extensive shock to the local community and tightened social distancing policy by the government [27]. Our study results show that the participants were experiencing high levels of fear (mean = 7.59/10), worries of getting infected (mean = 7.79/10), and perceived influences of COVID-19 (mean = 8.10/10) at that moment.

### 4.2. Depression, Anxiety, Stress, and Quality of Life

Moreover, we found that a considerable proportion of women participants were experiencing stress (32.2%), anxiety (42.4%), and depression (44.9%) during the ‘third wave’ in Hong Kong. The proportion is higher than a previous local study conducted in April 2020, in which 19%, 14%, and 25.4% of those respondents had depression, anxiety, and mental health deterioration [2]. Consistent with other studies [13,25,28], our study results also find significant negative correlations between emotional states (stress, anxiety, and depression) and different domains of WHOQOL (physical, psychological, social, and environmental). One of the studies investigated the relationships between physical and social-behavioral changes and the mental status of homebound young adults, which reported that 29.7% of respondents had moderate to severe depression, and a significant association was discovered between physical health and social contact changes with depressive symptoms [28]. Kwok and his team [25] deliberated that anxiety could provoke preventive measures but unfavorably disrupt daily work and family life if excessive, echoing our study results which show significant negative correlations between emotions and quality of life. As a comparison, our participants displayed lower mean scores in all four domains of quality of life (physical = 14.07; psychological = 12.72; social = 12.90; environmental = 12.24) than the mean scores of the abovementioned local study conducted in April 2020 [2], as well as the overall mean scores of the WHOQOL Group’s report in 23 countries (physical = 16.2; psychological = 15.0; social = 14.3; environmental = 13.5) [29]. This may imply that our study participants had lower quality of life.

### 4.3. General Self-Efficacy

According to Bandura [30], personal self-efficacy expectations will influence a person’s initiation in performing coping behaviors. In the present study, the GSE was found to be positively correlated with the four domains of life quality (physical, psychological, social, and environmental), reflecting that those with better quality of life have more confidence in coping with difficult life situations during COVID-19. Usually, people will participate in the activity and show self-confidence when they judge that they can handle the crises. If they think the crises beyond their ability to manage, they will worry about and avoid threatening situations [31]. Although Hong Kong seldom has large catastrophic disasters that require large-scale evacuation, a local study investigated the citizens’ perceived self-efficacy to prepare for disasters and barriers to decisions about disaster evacuation in 2017 [31]. The participants believed they could manage and were willing to comply with and engage in the related activities. In comparison, the citizens reported higher GSE mean scores (females’ mean = 24.52; males’ mean = 25.18) than our women participants who were experiencing the COVID-19 pandemic (females’ mean = 22.95, SD = 6.04). Our participants’ GSE scores were negatively correlated with their emotional states (stress, anxiety, and depression), which imply that those with adverse emotional conditions were not confident in dealing with the challenging situations during the pandemic. Therefore, the Hong Kong government and the local non-governmental organizations could consider strategies and provide more information and support to low self-confidence women in responding to public health emergencies.

### 4.4. Caregivers’ Roles

Women are the primary informal caregivers, with responsibilities including housework and childrearing within their families, especially in Asian countries and cities, including in Hong Kong. Among the 238 caregivers in our study, more than half were caregivers of both children and the elderly. Additionally, 87 caregivers had to care for two or more children, and 37 for two or more elderly people. Our results show significant differences in caregivers and non-caregivers regarding stress, anxiety, general self-efficacy, and the four quality of life domains. The caregivers had encountered adverse mental status, and perceived lower self-efficacy. Based on the local government’s population estimation, the average domestic household size is 2.7, which is smaller than the domestic household size in previous years [32]. Thus, the family caregivers, especially the women, might be responsible for caring for different generations. A local comparative population-based study reported that women, as primary caregivers of the elderly, were presented with the double risk of psychological and physical symptoms to a greater extent than non-caregivers, e.g., unstable emotions, anxiety, depression, chest discomfort, and dizziness [33]. A recent systematic review and meta-analysis of 74 studies found that subjective caregiver burden has a large positive association with informal caregiver anxiety [34]. The study findings suggest that early prevention and identification of subjective caregivers’ burden and anxiety with provision of psychological support and interventions to relieve their caring roles may be useful. Careful consideration of the caregivers’ physical and mental well-being with adequate rest and support to prevent physical exhaustion and psychological overwhelm during crises is recommended.

### 4.5. Low-Income Family and Low Education Attainment

The pandemic has caused immeasurable loss for the worldwide economy due to nationwide lockdowns and home confinement strategies that prohibit international and local economic activities. The global public health catastrophe has triggered an awful employment crisis [35]. Women account for more than half of the labor force in labor-intensive service industries globally, e.g., retail trade, hospitality, tourism, and healthcare, where remote work is usually not feasible [35]. Based on the recent employment statistics of the Census and Statistics Department of the Hong Kong Government [36], there has been an evident upsurge in unemployment and underemployment since the outbreak. As of January 2021, the unemployment and the underemployment rate were up to 7% and 3.8%, respectively. However, not just the consumption- and tourism-related sectors are affected; the labor market in other sectors is also worsening, such as, remarkably, in education and arts, entertainment, and leisure [36]. This study’s participants with low family income and low education attainment experienced significant psychological impacts. Most families might have extra household expenses to purchase personal protective and cleansing items, such as face masks and disinfectants. Moreover, due to the closure of schools and study centers, most families required an additional budget in purchasing computers or relevant facilities for their children’s online learning at home. All these expenses further intensified the financial and psychological burden, especially among lower-income families.

Local government, non-governmental organizations and healthcare organizations are suggested to establish activities that could support the women, especially those who are vulnerable or significantly affected during the COVID-19 pandemic. The activities enhance the self-confidence, physical, psychological, social, financial perspectives of the women and their life quality. Due to social distancing policy and rules limiting group gatherings, it is encouraged to organize flexible modes of activities, such as online/web-based seminars or workshops, telephone hotlines, and small-group/individual face-to-face consultations to provide physical and psychological support and well-being to those severely affected by appropriate healthcare professionals. For instance, courses or service-related healthy lifestyles, psychological first aid, and mental health first aid may be beneficial to the citizens. Accessible financial support for those citizens with lower household income or underemployment during COVID-19, e.g., anti-pandemic funds, is recommended. In addition, respite care services could be considered to relieve women’s stress and caregiving roles. Face-to-face individual or focus group in-depth qualitative interviews to understand women’s challenges and their needs, their life experience and their coping strategies in facing the pandemic are also recommended. In the long run, hopefully, the negative impacts of public health emergencies on the community could be lessened, and the women could be better supported in dealing with hardship in life events.

### 4.6. Limitations

Limitations must be considered when interpreting the study results, as there is potential bias. First, the study data were collected through online questionnaires completed by the women participants themselves, leading to concerns regarding objectivity in self-assessment. Additionally, an online self-reported questionnaire might lead to reporting bias or misunderstanding of the online questionnaire, although the participants were advised to contact the researchers if concerns arose. Those who do not have a smartphone, computer access, or sufficient knowledge regarding the Internet could not be included. However, due to social distancing during the pandemic, an online questionnaire to collect data was more feasible. The participants may not be representative of the general population. The online questionnaire was first disseminated to the Hong Kong Federation of Women’s Centres members and snowballed to others. Although these centers are located in different regions of Hong Kong and have members from different social communities, the demographics of the women participants may not be comparable and representative of the population data in Hong Kong. For instance, the age distribution of the study sample was similar to the population data but the household income was lower than the population census in the Hong Kong Census and Statistics Department [37,38]. Thus, the sample may not be the representative of the true population distribution. An appropriate randomized sampling method could be considered in a future study to reduce potential sampling bias.

## 5. Conclusions

The COVID-19 pandemic has impacted numerous countries worldwide in an inevitable manner. Although the Hong Kong government and citizens had previous experience responding to SARS and H1N1, the present study results indicate that the COVID-19 pandemic induced profound psychological and life impacts on women in Hong Kong. This study’s results show that those women who have a caregiver role, with lower income and low educational levels, require extra attention and support from the local government, non-governmental organizations, and healthcare organizations.

## Figures and Tables

**Table 1:** The participants’ mean score and standard deviation for the scales of DASS21, GSE, and WHOQOL-BREF (*n* = 417).

	Mean	SD	Range
DASS21			
Stress	12.35	8.82	0–21
Anxiety	7.71	7.76	0–21
Depression	9.35	8.40	0–21
GSE	22.95	6.04	10–40
WHOQOL-BREF			
Physical health	14.07	2.63	7–35
Psychological health	12.72	2.87	6–30
Social relationships	12.90	2.54	3–15
Environment	12.24	2.81	8–40

**Table 2:** Descriptive statistics of demographic variables, emotional states, general self-efficacy, and four domains of the quality of life (*n* = 417).

		**Stress**	**Anxiety**	**Depression**	**General Self-Efficacy**
	***n*** **(%)**	**Mean ± SD**	**Mean ± SD**	**Mean ± SD**	**Mean ± SD**
Age					
<50	212 (50.8)	14.4 ± 9.0	9.0 ± 8.3	10.3 ± 8.9	22.5 ± 6.5
≥50	205 (49.2)	10.2 ± 8.1	6.4 ± 6.9	8.4 ± 7.7	23.5 ± 5.6
Education					
Low	124 (29.7)	14.0 ± 9.1	9.8 ± 9.2	10.8 ± 9.5	21.3 ± 6.4
High	293 (70.3)	11.7 ± 8.6	6.8 ± 6.9	8.8 ± 7.8	23.7 ± 5.7
Married					
No	139 (33.3)	12.3 ± 8.4	7.9 ± 8.0	10.3 ± 8.7	23.0 ± 6.0
Yes	278 (66.7)	12.4 ± 9.0	7.6 ± 7.7	8.9 ± 8.2	23.0 ± 6.1
Caregiver					
No	179 (42.9)	10.3 ± 8.0	6.5 ± 7.3	8.7 ± 8.3	24.3 ± 6.1
Yes	238 (57.1)	13.9 ± 9.1	8.6 ± 8.0	9.8 ± 8.5	21.9 ± 5.8
Income					
<USD 2600	256 (61.4)	13.6 ± 9.3	8.9 ± 8.2	10.6 ± 9.0	21.8 ± 6.1
≥USD 2600	161 (38.6)	10.4 ± 7.7	5.8 ± 6.5	7.3 ± 7.0	24.8 ± 5.5
		**Physical Health**	**Psychological Health**	**Social Relationship**	**Environment**
	***n* (%)**	**Mean ± SD**	**Mean ± SD**	**Mean ± SD**	**Mean ± SD**
Age					
<50	212 (50.8)	13.6 ± 2.8	12.2 ± 3.0	12.7 ± 2.9	11.5 ± 3.0
≥50	205 (49.2)	14.6 ± 2.3	13.2 ± 2.6	13.2 ± 2.0	13.0 ± 2.4
Education					
Low	124 (29.7)	13.4 ± 2.7	12.1 ± 2.9	12.6 ± 2.6	11.2 ± 2.9
High	293 (70.3)	14.3 ± 2.6	13.0 ± 2.8	13.0 ± 2.5	12.7 ± 2.7
Married					
No	139 (33.3)	14.1 ± 2.8	12.4 ± 3.0	12.8 ± 2.7	12.3 ± 2.9
Yes	278 (66.7)	14.1 ± 2.6	12.9 ± 2.8	13.0 ± 2.5	12.2 ± 2.8
Caregiver					
No	179 (42.9)	14.7 ± 2.4	13.3 ± 2.8	13.3 ± 2.4	13.0 ± 2.7
Yes	238 (57.1)	13.6 ± 2.7	12.3 ± 2.8	12.6 ± 2.6	11.7 ± 2.8
Income					
<USD 2600	256 (61.4)	13.7 ± 2.8	12.4 ± 3.0	12.7 ± 2.7	11.8 ± 3.0
≥USD 2600	161 (38.6)	14.7 ± 2.2	13.3 ± 2.5	13.3 ± 2.2	13.0 ± 2.4

**Table 3:** Correlation table between emotional states, general self-efficacy, and the four domains of quality of life.

	Stress	Anxiety	Depression	General Self Efficacy	Physical Health	Psychological Health	Social Relationships	Environment
Stress		0.82	0.81	−0.46	−0.63	−0.62	−0.48	−0.54
Anxiety			0.83	−0.43	−0.62	−0.61	−0.48	−0.50
Depression				−0.46	−0.60	−0.68	−0.46	−0.47
General Self efficacy					0.58	0.69	0.60	0.57
Physical health						0.77	0.64	0.75
Psychological health							0.72	0.71
Social relationships								0.60
Environment								

All correlations are significant with *p* < 0.01.

**Table 4:** Bivariate regression models of emotional states, general self-efficacy and the four domains of quality of life.

	**Stress**	**Anxiety**	**Depression**	**General** **Self-Efficacy**
	**B(SE)**	**B(SE)**	**B(SE)**	**B(SE)**
Age				
<50 ^#^				
≥50	−4.21(0.84) ***	−2.57(0.75) **	−1.97(0.82) *	1.03(0.59)
Education				
Low ^#^				
High	−2.32(0.94) *	−3.03(0.82) ***	−2.00(0.90) *	2.39(0.64) ***
Married				
No ^#^				
Yes	0.04(0.92)	−0.22(0.81)	−1.36(0.87)	0.04(0.63)
Caregiver				
No ^#^				
Yes	3.64(0.86) ***	2.11(0.76) **	1.12(0.83)	−2.36(0.59) ***
Income				
<USD2600 ^#^				
≥USD2600	3.11(0.88) ***	−3.11(0.77) ***	−3.34(0.83) ***	2.96(0.59) ***
	**Physical Health**	**Psychological Health**	**Social Relationship**	**Environment**
	**B(SE)**	**B(SE)**	**B(SE)**	**B(SE)**
Age				
<50 ^#^				
≥50	0.99(0.25) ***	0.99(0.28) ***	0.49(0.25) *	1.48(0.27) ***
Education				
Low ^#^				
High	0.90(0.28) **	0.90(0.31) **	0.46(0.27)	1.43(0.29) ***
Married				
No ^#^				
Yes	−0.06(0.27)	0.43(0.30)	0.17(0.26)	−0.10(0.29)
Caregiver				
No ^#^				
Yes	−1.09(0.26) ***	−1.08(0.28) ***	−0.68(0.25) **	−1.34(0.27) ***
Income				
<USD 2600 ^#^				
≥USD 2600	0.96(0.26) ***	0.88(0.29) **	0.57(0.25) *	1.21(0.28) ***

B: unstandardized coefficients; SE: standard error of the regression coefficient; ^#^ reference group of categorical variables; * *p* < 0.05; ** *p* < 0.01; *** *p* < 0.001.

**Table 5:** Multivariable regression models of emotional states, general self-efficacy and the four domains of quality of life.

	**Stress ^^^**	**Anxiety ^^^**	**Depression ^^^**	**General** **Self-Efficacy ^^^**
	**B(SE)**	**B(SE)**	**B(SE)**	**B(SE)**
Age				
<50 ^#^				
≥50	−3.18(0.90) ***	−1.87(0.80) *	−1.61(0.88)	−0.02(0.62)
Education				
Low ^#^				
High	−0.93(0.96)	−1.99(0.85) *	−0.99(0.93)	1.43(0.65) *
Married				
No ^#^				
Yes	−0.27(0.91)	−0.35(0.81)	−1.22(0.88)	0.31(0.62)
Caregiver				
No ^#^				
Yes	2.03(0.94) *	0.92(0.83)	0.25(0.91)	−1.97(0.64) **
Income				
<USD 2600 ^#^				
≥USD 2600	−2.34(0.90) *	−2.28(0.80) **	−2.81(0.88) **	3.00(0.59) ***
	R^2^ = 0.095 ***	R^2^ = 0.077 *	R^2^ = 0.055 **	R^2^ = 0.095 ***
	**Physical Health ^^^**	**Psychological Health ^^^**	**Social** **Relationship ^^^**	**Environment ^^^**
	**B(SE)**	**B(SE)**	**B(SE)**	**B(SE)**
Age				
<50 ^#^				
≥50	0.61(0.27) *	0.58(0.30) *	0.228(0.269)	1.06(0.28) ***
Education				
Low ^#^				
High	0.52(0.29)	0.59(0.31)	0.25(0.29)	0.96(0.30) **
Married				
No ^#^				
Yes	0.07(0.27)	0.60(0.30) *	0.27(0.27)	0.05(0.28)
Caregiver				
No ^#^				
Yes	−0.72(0.28) *	−0.83(0.31) *	−0.56(0.28)	−0.72(0.29) *
Income				
<USD2600 ^#^				
≥USD2600	0.67(0.27) *	0.51(0.30)	0.39(0.27)	0.77(0.28) **
	R^2^ = 0.085 ***	R^2^ = 0.078 ***	R^2^ = 0.032 *	R^2^ = 0.144 ***

B: unstandardized coefficients; SE: standard error of the regression coefficient; ^#^ reference group of categorical variables; ^^^ adjusted for age, education, married or not; * *p* < 0.05; ** *p* < 0.01; *** *p* < 0.001.

## Data Availability

The data presented in this study are available on reasonable request from the corresponding author.
